# The Candler Bill

**Published:** 1887-08

**Authors:** 


### THE CANDLER BILL.

It will be gratifying intelligence to the entire medical profession in Georgia, as it ought to be to all sensible people, to know that the Legislature has finally passed the bill indicated in the caption of this article. The bill itself was published some months ago in THE JOURNAL, and our readers will no doubt remember its main features. These are, first, a detailed scheme for turning over to the profession a certain class of dead bodies for dissection, and fairly distributing them among the various medical colleges; second, changing the crime of grave-robbing from mere misdemeanor to felony.

It is really remarkable that it has taken so long a time to convince Legislatures that the most effective way to prevent this offense is by providing a lawful method for doing that which all sensible people know must be done. The bill as passed is satisfactory to the profession, and a careful perusal will show that it thoroughly guards the rights of the public, and will protect all persons against the danger of having those near or dear to them passing into the dissecting-room. Neither the desecration of graves nor the enforced surrender of the bodies of friends is possible any longer.

In the face of the superstition and bigotry which have been shown in the past on this subject, the good sense of Mr. C. M. Candler, of DeKalb, the father of this bill, stands very conspicuous. To him is due the credit, not only of drafting this very sensible and well-guarded law, but also of engineering it to a successful passage. Observing the character of the violations of the old law, Mr. Candler very properly concluded that the true remedy was not in senseless tirades, but in a rational law which would remove the cause of grave-robberies; hence his statesman-like bill. And here we feel constrained to say that, although a very young man, Mr. Candler has not only in this matter, but in his entire course in the Legislature, shown such rare good sense, such industrious, business-like, intelligent attention to the duties of his position, that we earnestly hope he will be continued in office. It will be a real benefit to the State if other counties will send up as good men.

The Journal modestly claims for itself the credit of being the only medical publication in the State that has done anything toward furthering the passage of this law. Our readers well remember that we have been for a long time urging this measure upon successive Legislatures. We have had able and timely assistance in this instance from the *Evening Capitol*, *Evening Journal*, *Constitution*, the *Wesleyan Christian Advocate*, published at Macon, and the *Christian Index*, of Atlanta. The profession owe considerable gratitude to these papers.

